# Multicancer Early Detection Tests: A State‐of‐the‐Art Review for Otolaryngologists

**DOI:** 10.1002/oto2.70040

**Published:** 2024-10-26

**Authors:** Elena Kennedy, Greg Durm, Janice L. Farlow

**Affiliations:** ^1^ Department of Otolaryngology–Head and Neck Surgery Indiana University School of Medicine Indianapolis Indiana USA; ^2^ Department of Medicine, Division of Hematology/Oncology Indiana University School of Medicine Indianapolis Indiana USA

**Keywords:** biomarker, cancer screening, head and neck cancer, liquid biopsy, multicancer early detection tests

## Abstract

**Objective:**

To provide a review of the science and applicability of current multi‐cancer early detection (MCED) tests for otolaryngologists.

**Data Sources:**

PubMed, clinicaltrials.gov, company websites.

**Review Methods:**

Using PRISMA methodology, primary literature regarding MCED tests was queried from April 26 to May 12, 2024 using MCED search terms. Ongoing clinical trials incorporating MCED screens were identified via the National Institutes of Health clinicaltrials.gov website. Company websites for available or upcoming MCED tests were reviewed.

**Conclusion:**

Long‐term robust data regarding the performance characteristics, effects on clinical outcomes, and cost‐utility of MCED tests for head and neck cancer are currently lacking. Otolaryngologists should be aware of the implications of MCED tests as these assays become more widely used.

**Implications for Practice:**

Although not FDA‐approved or covered by insurances at the time of writing of this manuscript, MCED testing is rapidly gaining interest, and patients with positive tests are presenting to otolaryngologists for evaluation. While MCED technologies hold great promise for early detection of disease and potential reduction of morbidity and mortality, more study is needed about their utility for head and neck cancer and optimal diagnostic workflows.

Cancer is the leading cause of death globally and the second leading cause of all deaths in the United States.^1^, ^2^ Screening and prevention efforts have had a significant impact on the nationwide incidence of prostate, breast, lung, and cervical cancer. Since the 1990s, cancer death rates have decreased by nearly 32%, much of which is attributed to early screening, detection, and prevention measures.^3^, ^4^, ^5^, ^6^ Furthermore, it is estimated that cancer screening has saved at least 6.5 trillion dollars by treating cancer at earlier stages of presentation.^7^, ^8^

Unlike many other solid tumor sites, head and neck cancers represent a diverse group of malignancies without widely implemented screening. Altogether, head and neck malignancies are the seventh most common cause of cancer globally.^9^ Head and neck squamous cell carcinoma (HNSCC) is by far the most common, accounting for nearly 450,000 deaths yearly.^10^, ^11^ Although the decrease in tobacco use led to an initial decrease in incidence for developed countries, HPV‐positive oropharyngeal SCC are accounting for an overall increasing incidence worldwide.^12^, ^13^ No validated and recommended screening tests exist beyond a routine history and physical examination. Delays in referrals to a head and neck specialist, coupled with generally late presentations of disease, contribute to many head and neck cancers being diagnosed at advanced stages.^10^ Thus, the recent development of multi‐cancer early detection (MCED) tests, some which may also screen for head and neck cancers, has been met with great interest.

MCED tests test for biomarkers of a variety of different tumors at once. As “liquid biopsies,” they involve collection of blood, urine, saliva, stool, or other biomedical samples to be tested for entire cells, proteins, nucleic acids, or other analytes shed by tumors (Figure 1). Cancer is suggested by the presence of these analytes if unique to cancers like tumor‐specific antigens, or additional analysis for genomic, transcriptomic, epigenomic, or other alterations. If there is a cancer signal detected, MCED tests can use the specific extractable information from these analytes to identify possible sites of origin. Each type of analyte can provide different information (eg, circulating tumor cells can be analyzed for genomic, transcriptomic, and metabolic data; while circulating tumor DNA only provides DNA), but differs in its practicality of application to specific cancer types (eg, some cancers have very few circulating tumor cells or specific proteins for enrichment).^14^ The ease of administration of MCED tests as compared to tissue biopsies and application to a broader population create the opportunity to identify head and neck cancer at earlier, asymptomatic stages and streamline referrals to head and neck specialists. At the time of this manuscript, there are no current FDA‐approved MCEDs, although some are available through health systems via direct purchase by consumers. Despite their relative rarity and expense, based on our experience and personal communication with colleagues at other tertiary academic medical centers, MCEDs are increasing referrals to otolaryngologists for positive tests. To date, however, there are no reviews or guidelines regarding MCEDs specific to otolaryngologists. Thus, we sought to perform a state‐of‐the‐art review to survey the evidence base behind common MCEDs, as well as upcoming relevant studies.

**Figure 1:**
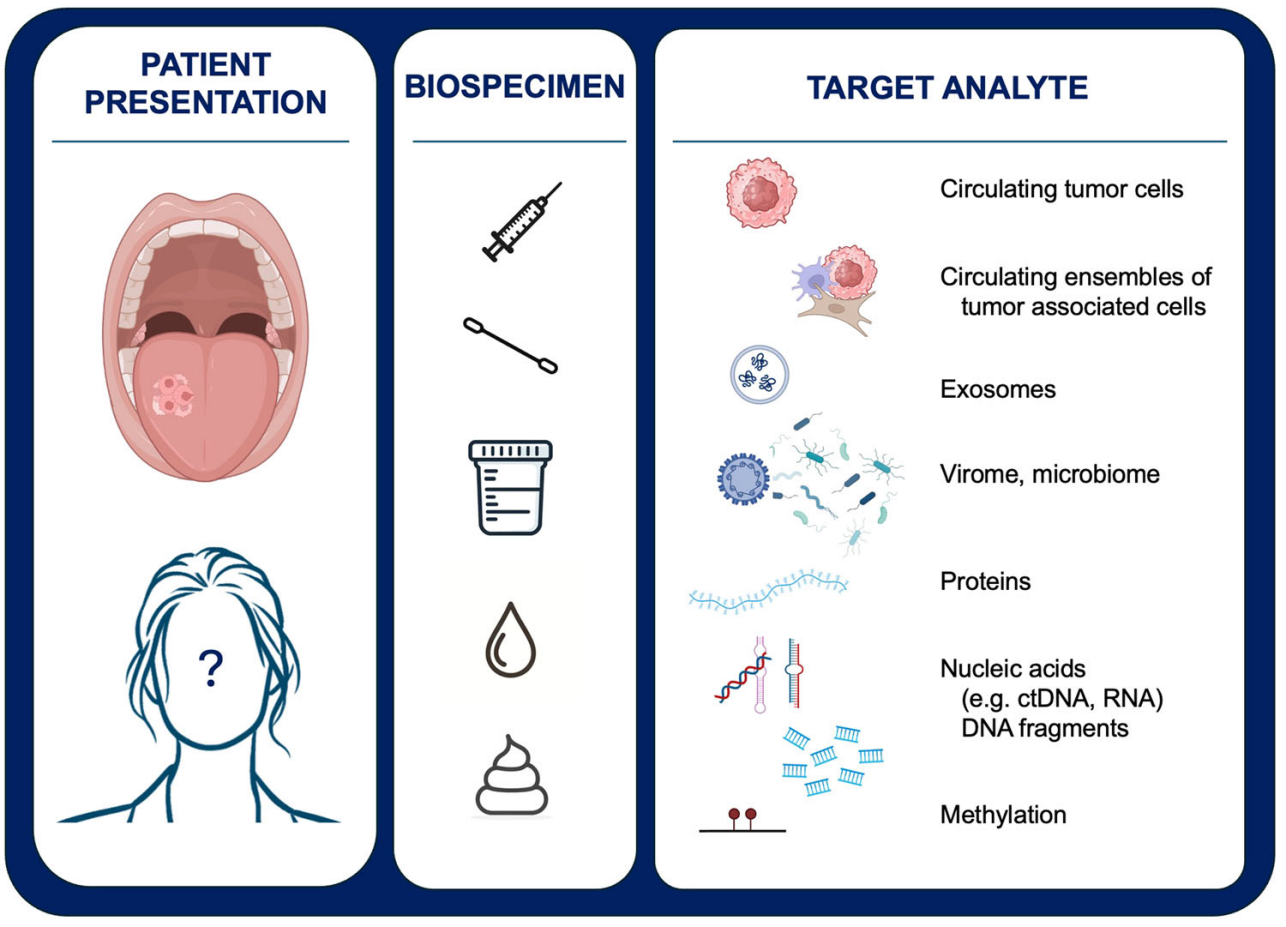
Multi‐cancer early detection tests. Assays currently available and in development can be used for both symptomatic/at‐risk or asymptomatic individuals. A variety of biospecimens can be tested including blood, saliva, urine, tears, and stool. Tests analyze a specific type of target or may integrate signals detected from a variety of analytes that suggest the presence of a cancer. Images generated with ChatGPT v4.0 and BioRender.

## Methods

Following Preferred Reporting Items for Systematic reviews and Meta‐Analyses (PRISMA) methodology,^15^ PubMed was queried between April 1 and May 12, 2024. Search terms used to find relevant articles included “multi‐cancer early detection” or “multi‐cancer detection.” Studies were excluded if they were editorials, reviews, systematic reviews, meta‐analyses, Delphi consensus studies, not in English, or not in humans. Publications involving the same clinical trial were reviewed, and a representative study was chosen. Studies specifically mentioning head and neck cancers were separately tagged. Studies were independently screened by 2 authors (EK and JLF) for inclusion. References of included articles were reviewed for additional studies. Extracted data included the study location and type, the study target, cancer types evaluated, and study sample sizes. Given the variety of studies including but not limited to small focus‐group studies, mathematical modeling analyses, and prospective randomized controlled trials, qualitative data synthesis without use of a formal bias assessment tool was performed by all authors. To supplement the review of the current published literature, technology and screening tests found in relevant peer‐reviewed articles were further explored on company websites. Ongoing clinical trials including “multi‐cancer early detection” or “multi‐cancer detection” were obtained using the National Institutes of Health clinicaltrials.gov search engine, which was queried on May 12, 2024.

## Discussion

### Evidence base behind current multi‐cancer early detection tests

**Figure 2:**
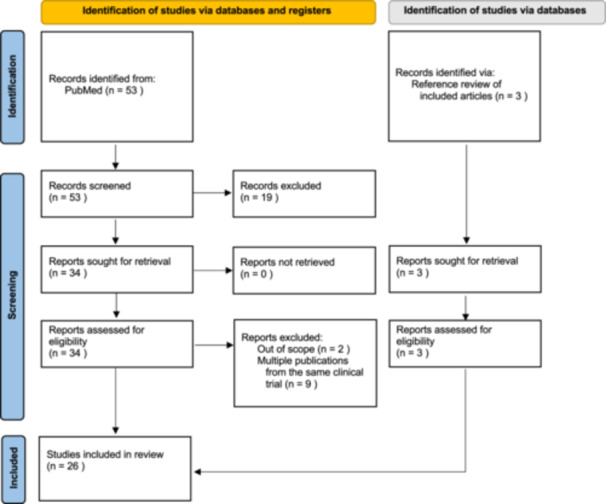
Preferred Reporting Items for Systemic Reviews and Meta‐Analyses (PRISMA) diagram of included studies.

There is limited primary research into MCED tests, with a total of 26 independent studies included in this review, the majority of which were published in 2022 or later (Figure 2, Supplemental Table S1, available online). The available research encompassed a wide variety of study designs, including the evaluation of the performance characteristics of various MCED assays, cost‐utility analysis, modeling population screening, and qualitative research regarding patient preferences for multicancer screening tests. To date, only 2 known MCED tests, Galleri (GRAIL) and Trucheck's Intelli MCED (Datar Cancer Genetics) are clinically available and include screening for head and neck cancers (Table 1). Although neither are currently FDA‐approved, they can be purchased through a physician or directly by patients.

**Table 1:** Brief Overview of Common Multi‐Cancer Early Detection Tests

Test name (Company)	Detection method	Sensitivity overall (Head and neck cancer)	Specificity overall (Head and neck cancer)	Current cost (USD)
Galleri (GRAIL)	Cell‐free DNA methylation	30%‐90% by stage (86%)	99% (71%‐83%)	$949
Trucheck Intelli (Datar Cancer Genetics)	Circulating tumor cells	60%‐90% (93%)	>95% varying by cancer types (98%)	$1142
OverC (Burning Rock)	Cell‐free DNA methylation	70%‐75% (N/A)	95%‐99% (N/A)	Unknown
CancerSEEK/CancerGUARD (Exact Sciences)	Cell‐free DNA with protein biomarkers	69%‐98% by stage (N/A)	99% (N/A)	$500
OneTest (20/20 GeneSystems)	Tumor antigens	60%‐80% (N/A)	80% (N/A)	$39‐189
MiRAM (Elypta)	Glycosaminoglycans	43%‐62% (N/A)	95%‐99% (N/A)	<$50

Sensitivities and specificities of available multi‐cancer early detection tests. Currently, only Galleri and Truckeck Intelli are validated for detection of head and neck cancer.

Galleri is a custom‐targeted methylation panel based on research that has identified cancer‐ and/or tissue‐specific methylation patterns in cell‐free DNA, particularly of certain tumor suppressor genes.^16^ Through a variety of research studies, mainly large prospective case‐control or cohort studies, Galleri has analyzed over 50 different cancer types with a high specificity and sensitivities ranging from 30% to 90% across all cancers.^16^ The test currently defines “head and neck” as a single cancer signal origin, within which cancers of the oropharynx, hypopharynx, nasopharynx, larynx, lip and oral cavity, nasal cavity, paranasal sinuses, and major salivary glands are grouped. Separate cancer signal origins tested for that can still relate to the head and neck include bone and soft tissue (sarcomas), neuroendocrine cells (Merkel cell), and the thyroid gland, or potentially other malignancies that can metastasize to the cervical lymph nodes. Within their group of head and neck cancers, they demonstrate an average sensitivity of 85.7% (95% CI 77.8%‐91.1%) across cancer stages (63% for Stage I up to 96% for Stage IV) and subtypes (highest sensitivities for oropharynx and nasopharynx cancers).^17^ Specificities for grouped head and neck cancers were reported from 71% to 83%.^15^ Of note, the sensitivity of Galleri for other cancer signal origins that may relate to the head and neck are all less than 70%, with the next highest sensitivity for sarcomas at 60%. Patients and providers receive a report that gives a binary outcome (cancer signal detected or not), as well as up to 2 predicted tissue or organ type that serve as the origin of the cancer (cancer signal origin). With a predicted cancer signal origin, a shaded bar is also shown to give a visual approximation of the match of the DNA methylation pattern to cancers of that tissue or organ, but this not quantified as a percentage, nor are details of specific methylation profiles delineated. Although not currently FDA‐approved, Galleri is available in the United States for an out‐of‐pocket cost of about $949.^18^

Current data available for the societal impact of Galleri is not specific to the head and neck and is generalized for this MCED. Mathematical modeling based on the Galleri test use in the United Kingdom suggests that there could be a 46% reduction in mortality with MCED, driven largely by a reduction in late‐stage diagnosis.^19^ Of note, this translates to an absolute reduction from 13 individuals per 100,000 persons who would die with usual care, to 7 individuals per 100,000 persons who would die with widespread implementation of MCED screening. The investigators subsequently applied their model to areas in the United Kingdom with varying levels of socioeconomic deprivation; they found that reductions in cancer mortality for head and neck cancers would likely be higher in areas of higher deprivation.^20^

Trucheck's Intelli is the other prominent MCED test known to detect around 70 different types of solid tumors including various cancer types in the head and neck.^21^ The assay detects and enriches for circulating ensembles of tumor‐associated cells (C‐ETAC). In their proof of concept study, C‐ETACs were identified in 91.7% of previously diagnosed head and neck cancers (n = 1361), 88.3% of thyroid cancers (n = 60), and 84.4% of skin cancers (n = 32).^22^ They also found no significant differences between detection rates in therapy naïve and pretreated individuals, or between metastatic and non‐metastatic cases.^22^ Cancer types relevant to the head and neck and their associated markers used in the Intelli test include “head and neck” (p63, HMWCK, CK5/CK6), “thyroid” (TTF‐1, thyroglobulin, calcitonin, CK19), and “sarcoma” (SMA, S100, CSV).^21^ In combined retrospective and prospective data, the test had a reported sensitivity of 92.5% and organ‐specific accuracy of 98.3% for “head and neck cancers.” Sarcoma sensitivity was 95.1% and 100% organ‐specific accuracy, while thyroid cancers had 100% sensitivity and 100% organ‐specific accuracy. The results of the test for each cancer subtype are listed as either positive (circulating antigen present), negative (lack of circulating antigen), or indeterminate (circulating antigen is present but tissue of origin or type of cancer could not be detected).^21^, ^22^, ^23^ Although not currently available in the United States, this test is available for purchase in the United Kingdom at around $1142 USD.^18^

We did not identify any published MCED studies focused specifically on head and neck cancer patients. To our knowledge, there is only 1 published retrospective study on MCEDs in head and neck cancer. The head and neck surgery group at Mayo Clinic reviewed 5 patients who came to their clinic with positive test results from commercially obtained Galleri.^24^ Of these patients, 2 patients were diagnosed with p16+ oropharyngeal squamous cell carcinoma, 1 was diagnosed with sarcoma of the thigh, and the remaining 2 did not have a malignant tumor identified. Galleri had suggested a possible head and neck primary in all patients except for one of the patients who did not end up finding a cancer. Their workup for these patients included a thorough physical exam, flexible nasopharyngoscopy, and cross‐sectional imaging. Identified tumors can proceed with standard‐of‐care treatment; however, next steps are challenging when there are no abnormalities on physical exam, scope, or imaging. In this situation, the Mayo Clinic group recommends obtaining PET/CT scan, with maintenance surveillance (6–12 months) if the scan is negative. Other studies suggested similar diagnostic workflows following MCED test suggesting the presence of head and neck cancer.^25^

### Ongoing and Future Research for MCEDs Across Cancer Types

When combined with data available on clinicaltrials.gov, our review identified a total of 30 relevant clinical trials (Table 2). The Multicancer Early Detection Consortium, created in 2022 to align United States and United Kingdom efforts for evaluation of and guidance for MCED technologies, has helped coordinate at least a dozen of these studies.^26^ The majority of trials focus on evaluating methylation markers in peripheral blood samples, although other genomic, transcriptomic, and proteomic targets, or even the complementary use of multiple analyte types, in both blood and urine are being studied. Additional studies and multicenter initiatives also exist to understand health equity and policy implications of MCEDs.

**Table 2:** Ongoing or Upcoming Clinical Trials Related to Multi‐Cancer Early Detection Tests

Study/type	Acronym/short title	Title	Location	Test (sponsor)	Target analytes (biospecimen type)	Estimated completion
NCT04241796 Cohort	PATHFINDER	Assessment of the Implementation of an Investigational Multi‐Cancer Early Detection Test Into Clinical Practice	USA	Galleri (GRAIL)	Methylation (blood)^a^	2022
NCT05611632 RCT	NHS‐Galleri	Does Screening With the Galleri Test in the NHS Reduce the Likelihood of a Late‐stage Cancer Diagnosis in an Asymptomatic Population	UK	Galleri (GRAIL)	Methylation (blood)^a^	2026
NCT05205967 Cohort	REFLECTION	A Clinical Practice Learning Program for Galleri	USA	Galleri (GRAIL)	Methylation (blood)^a^	2026
NCT05155605 Cohort	PATHFINDER2	A Multi‐Cancer Early Detection Study	USA, Canada	Galleri (GRAIL)	Methylation (blood)^a^	2027
NCT05673018 Cohort	REACH	Real‐world Evidence to Advance Multi‐Cancer Early Detection Health Equity	USA	Galleri (GRAIL)	Methylation (blood)^a^	2030
NCT03934866 Cohort	SUMMIT	A Cancer Screening Study	UK	Galleri (GRAIL)	Methylation (blood)^a^	2030
NCT05235009 Case‐control	LEV87A	GAGomes for Multi‐Cancer Early Detection in Asymptomatic Adults	Sweden	MIRAM (Elypta)	Metabolome (blood, urine)^a^	2025
NCT05295017 Cohort	LEV93A	GAGomes for Multi‐Cancer Early Detection in High‐Risk Adults	UK	MIRAM (Elypta)	Metabolome (blood, urine)^a^	2025
NCT05780957 Cohort	LEV65	Multi‐Cancer Early Detection of Firefighters	US	MIRAM (Elypta)	Metabolome (blood, urine)^a^	2030
NCT04820868 Case‐control	THUNDER	The Unintrusive Detection of EaRly‐stage Cancers	China	OverC (Burning Rock)	Methylation (blood)^a^	2022
NCT04822792 Case‐control	PRESCIENT	Pan‐canceR Early‐Stage deteCtion by lIquid Biopsy tEchNique projecT	China	OverC (Burning Rock)	Methylation, protein (blood)^a^	2023
NCT04817306 Case‐control	PREDICT	Pan‐canceR Early DetectIon projeCT	China	OverC (Burning Rock)	Methylation (blood)^a^	2023
NCT04383353 Case‐control	PREDICT	Pan‐canceR Early DetectIon projeCT	China	OverC (Burning Rock)	Methylation (blood)^a^	2023
NCT05227534 Cohort	PREVENT	Multi‐canceR Early‐detection Test in Asymptomatic Individuals	China	OverC (Burning Rock)	Methylation (blood)^a^	2028
NCT04972201 Case‐control	PROMISE	A Proof of Concept Study of Pan‐cancer Early Detection by Liquid Biopsy	China	(Chinese Academy of Medical Sciences)	Methylation, mutations, miRNA markers (blood)	2022
NCT04903665 Case‐control	PERCEIVE‐I	PERformance of Multi‐Cancer Early‐detectIon Based on Various Biomarkers in fEmale Cancers	China	(Wu, Fudan University)	Methylation, mutations, miRNA markers (blood)	2022
NCT04835675 Case‐control	ASCEND‐Hep	AssesSment of Early‐deteCtion basEd oN liquiD Biopsy in Hepatobiliary Cancer Malignancies	China	(Zhujiang Hospital)	Methylation, RNA markers (blood)	2022
NCT05431621 Case‐control	N/A	Establishment of Molecular Classification Models for Early Diagnosis of Digestive System Cancers	China	(Singlera Genomics)	Methylation, fragmentomics, miRNA markers, circulating tumor cells (blood)	2023
NCT05685524 Case‐control	PanCa	Development of Diagnostic Model for Multi‐cancer Diagnosis Based on DNA Methylation Biomarkers	China	(Wuhan Ammunition Life‐tech)	Methylation (blood)^a^	2023
NCT05495685 Case‐control	DAYBREAK	iDentification and vAlidation Model of Liquid biopsY Based cfDNA Methylation and pRotEin biomArKers for Pancreatic Cancer	China	(ShiWei, Changhai Hospital)	Methylation, protein, miRNA markers (blood)	2024
NCT05874648 Case‐control	PROFUTURE	PRediction Of Five Usual Tumors Using Blood Test for Risk Assessment and Early Detection	China	(Sun Yat‐sen University)	Methylation, fragmentomics, protein (blood)	2024
NCT06001099 Case‐control	PERCEIVE‐II	PERformance of Multi‐Cancer Early‐detectIon Based on Various Biomarkers in fEmale Cancers	China	(Zheng, Fudan University)	Methylation, mutations, miRNA markers (blood)	2024
NCT06391749 Cohort	K‐ACCELERATE	Clinical Validation of an MCED Test in Symptomatic Populations	Vietnam	SPOT‐MAS (Gene Solutions)	Methylation, fragmentomics, copy number variation (blood)	2025
NCT05334069 Case‐control	Alliance Biobank Study	Collecting Blood Samples From Patients With and Without Cancer to Evaluate Tests for Early Cancer Detection	USA	(Alliance for Clinical Trials in Oncology)	To be determined (blood)^a^	2025
NCT05633342 Cohort	CADENCE	CAncer Detected Early caN be CurEd	Singapore	(MiREX)	Methylation, RNA, miRNA, protein (blood)	2025
NCT05366881 Case‐control	CAMPERR	cfDNA Assay Prospective Observational Validation for Early Cancer Detection and Minimal Residual Disease	USA	(Adela)	Methylation (blood)^a^	2026
NCT04825834 Case‐control	DELFI‐L101	DNA Evaluation of Fragments for Early Interception ‐ Lung Cancer Training Study	USA	(Delfi Diagnostics)	Fragmentomics (blood)^a^	2026
NCT06231953 Cohort	N/A	A Prospective, Multi‐center Clinical Study to Establish Multi‐Cancer Early Detection Platform Through the Analysis of Whole Genome Sequencing of Circulating DNA in Cancer Patients and Healthy Volunteers	Korea	(Yonsei University)	Genome (blood)	2026
NCT06217900 Case‐control	PROFOUND	a PROspective Case Control Study to Develop and Validate a Blood Test FOr mUlti‐caNcers Early Detection	China	(Weihe Medical Laboratory)	Methylation (blood)	2027
NCT06011694 Cohort	N/A	The Jinling Cohort	China	CanScan (Geneseeq, Nanjing Shihejiyin Technology)	Genome, fragmentomics (blood)	2027

Abbreviation: RCT, randomized controlled trial.

^a^ Denotes that head and neck cancers are advertised to be captured within the assay of interest.

While the intent of this review is not an exhaustive compilation of test characteristics for all MCED technologies, a few are highlighted here. At the time of writing this manuscript, Galleri was 1 of 3 companies with a MCED test that had received a Breakthrough Devices Designation by the FDA, which permits expedited review through the regulatory agency prior to approval. Galleri and Trucheck Intelli MCED tests are the focus of this review as they specifically include malignancies of the head and neck, but the following tests are described to show the variety of MCED tests closest to public use that can extended to use in head and neck cancer.

The other tests that received FDA Breakthrough Devices Designation include OverC and CancerSEEK/CancerGUARD (Exact Sciences). OverC, like Galleri, focuses on analyzing methylation data, with studies suggesting a sensitivity of 70% to 75% and specificities of 95% to 99%.^27^ We could not find estimates of cost for this test once available to the public. CancerSEEK utilizes both circulating tumor DNA (ctDNA) and protein biomarkers to detect cancer with overall sensitivity ranging from 69% to 98% and specificities of 99%.^28^, ^29^ The test currently is limited to fewer cancer types to lessen costs of the test and to minimize false positives. When commercially available, the makers estimate the test will cost around $500.^28^

Other MCED tests are focused on nongenomic biomarkers. OneTest (20/20 GeneSystems) measures different tumor antigens, such as prostate‐specific antigen, cancer antigen 125, and alpha‐fetoprotein for prostate, ovarian, and liver cancer respectively. It has reported specificities around 80% and sensitivities 60% to 80%, and is currently offered on the market between $39 and $189 depending on the clinical setting.^18^, ^30^ While technically the test has picked up oral cancer, they do specifically note poor sensitivity for head and neck cancer, particularly in early stages of disease.^30^ MiRAM (Elypta), meanwhile, focuses on glycosaminoglycan profiles, part of a patient's metabolomic profile, that can be ascertained in blood or urine. Initial case‐control studies identified sensitivities of 43% to 62% and specificities of 95% to 99%.^31^ Of note, the investigators predict that costs associated with the assay are significantly less than circulating free DNA‐based tests, with projected costs less than $50 a sample. They also suggest that their test may be particularly useful as not all cancer types result in significant DNA shedding into the peripheral blood, which is required for many of the other assays under development.

Given the significant interest in and population‐wide implications of MCEDs, organizations aiming to coordinate efforts across multiple institutions and companies have also joined the research landscape. The Alliance for Clinical Trials in Oncology, for example, is currently recruiting subjects to donate blood samples specifically to build a biobank that can be used to evaluate MCED tests (NCTN05334069). Additionally, the National Cancer Institute has initiated efforts to synergize and compare research efforts for multi‐cancer screening assays. A Cancer Screening Research Network (CSRN) was formalized in January 2024, in order to launch an NCI‐sponsored clinical utility randomized trial termed the Vanguard Study.^32^ The study will randomize patients to standard‐of‐care cancer screening versus separate arms with different MCED tests, in order to assess patient adherence, diagnostic workflows, and barriers to implementation in diverse populations. Studies like this are necessary both for head‐to‐head comparison of MCED tests, as well as to understand how best to implement any MCED paradigm. In the future, adaption of MCED into clinical practice may be based on a combination of multiomic technologies built from existing platforms, and perhaps targeted toward specific populations based on patient demographics, comorbidities, family cancer history, symptom profiles, or other risk factors.

### Caveats Regarding MCED Within Otolaryngology

MCED is exciting to many clinicians as they have the potential to detect multiple cancers at early stages, thus possibly improving cancer‐specific mortality and decreasing the physical, emotional, psychologic, and financial burden of a late‐stage cancer diagnosis. However, there are some concerns that arise with these advancements. Current data are early, and the tests have not been validated within the general population or with patients at risk for or diagnosed with head and neck cancer. Additionally, evidence‐based guidelines for follow‐up of positive MCED results are lacking, which raises concern of excessive, costly, psychologically distressing, or even inappropriate additional workup and interventions. Lastly, there are concerns about equitable access to these tests and value for society as a whole.

The first concern about MCED is that they likely overestimate positive predictive value, or the likelihood that a patient with a positive test truly has cancer. Its value is directly related to the prevalence of disease within a population and is key when considering the justifiability of a widely employed population screening test. Many of the studies used for preliminary validation of MCEDs use case‐control or cohort studies, in which the patients either had a known cancer diagnosis or were at risk/symptomatic. This results in skewing of the results in which there is an overestimation of positive predictive value compared to the actual general population. Furthermore, patients with diagnosed head and neck cancers have only represented a small percentage of subjects involved in published MCED studies.

Second, unlike the clear guidelines outlined for workup following positive traditional screening for breast, cervical, lung, or colorectal cancer, MCED tests do not currently tell clinicians what to do with a result that at best only suggests the implicated tissue of origin. Physicians are left to their best judgement and interpretation of the screening test. Despite promising preliminary data, there is still significant room for both false positive and false negative tests. When a patient has a positive screening test, often multiple physicians are involved in attempting to identify a potential cancer. The more cancers screened, the more challenges arise with specificity and determining tumor origin.^33^ Specifically important for the otolaryngologist is the challenge of detecting the exact tissue of origin for HPV‐mediated tumors, which can arise in multiple body sites including cervix, anus, and head and neck.^15^, ^17^, ^34^ Otolaryngologists may be involved at any point in the diagnostic workflow—either early if the MCED test suggests a head and neck primary, or later if the screening results are tissue‐agnostic or other workup has been negative. Thus, patients presenting to otolaryngologists may or may not exhibit symptoms, have cross‐sectional imaging with or without suspicious lesions, or have any visible or palpable lesions. Should a head and neck primary not be apparent on history, physical examination, in‐office endoscopic examination, or radiography, the otolaryngologist is left to determine if more invasive, costly, and potentially harmful workup such as sedated panendoscopy or surveillance imaging is needed. Without any risk stratification to the results, the surveillance is left to the discretion of the physician and patient.^18^, ^24^

There are also concerns that positive signals may arise from indolent tumors, where the tumor type may never advance to the point where intervention would alter cancer‐specific morbidity or mortality. As seen in Trucheck Intelli prospective testing of around 10,000 asymptomatic participants, they report a 230 times higher 1‐year cancer risk in patient who test positive for circulating tumor antigens. However, this resulted in only 10 new clinical cancer diagnoses within 1 year of follow‐up, thus leaving 250 people who tested positive on the MCED without a cancer found in that year.^23^ It remains unclear if those who tested positive and were not found to have a cancer had a small disease burden too small to be detected clinically, or if these represent false positive results. Additionally, for those patients with positive tests that were found to have a cancer clinically, it is not known if this early detection impacts patient oncologic, functional, or quality of life outcomes. These tests are prone to lead time bias where survival after an early detection test is long, but purely because of the time at which the cancer was detected, and not because of any benefit that early detection or treatment may have on survival itself. Collectively, this may lead to a large amount of unnecessary medical expenditure and harmful exposures without changing clinical outcomes.

In the same way, a positive finding can be difficult to interpret currently, a negative result may not be completely reassuring. Many MCED tests showed significantly increased sensitivities with late‐stage cancers. For example, ctDNA tends to be more easily detected with higher tumor burden and disease severity, thus testing has increased sensitivity at later stages.^18^ A negative MCED screening could indicate that a tumor is too early to be detected, as it does not produce a measurable amount of target analyte of the test. Furthermore, not all late‐stage tumors may shed molecules that are covered in liquid biopsy assays. It is still unknown whether the false reassurances in these situations would further delay diagnostic workup of symptomatic patients or compromise future evaluation of individuals at high risk for development of head and neck cancer.

Finally, there are profound societal implications of screening tests that must be considered. Those patients who have information about MCED and the financial resources to obtain a test are often not the same individuals who could benefit most from a screening test.^35^ Whether it be related to healthcare access, education, or other socioeconomic barriers, it has been well‐described that those of lower socioeconomic status, racial minorities, and rural patients have the lowest participation in cancer screening programs and often have worse cancer‐specific survival.^36^, ^37^ Furthermore, while the idea of MCEDs is generally well‐received, preliminary studies have shown significant differences in patient preferences for these screening tests, and these variations can stratify by health status, race, educational background, and socioeconomic classes.^38^, ^39^ It is plausible that vulnerable populations, some of whom have distrust of invasive testing and healthcare systems, may be more willing to utilize MCEDs if affordable. Currently, however, MCEDs are expensive and thus will likely be most accessible to those with more financial resources for the near future. The diversity of healthcare systems further complicates the acceptability, cost, and equity of incorporating MCED screening tests globally.

## Implications for Practice

Although no MCED tests are currently covered by insurance or incorporated into national screening guidelines, some are still available to patients. It is critical that otolaryngologists—who are the natural recipient of referrals for positive MCED tests suggesting a head and neck primary—be aware of the general types of tests available, as well as major caveats required in the interpretation of these tests. MCED testing holds great promise for cancer screening and earlier detection of disease, which could potentially translate to reduction in cancer‐specific morbidity and mortality. However, data available are sparse, particularly as it relates to head and neck cancer, and thus otolaryngologists should convey to interested patients that these technologies are still early in development and have risks of false positives/negatives that can lead to unnecessary workup or false reassurance. Physicians must employ shared decision‐making and consider patient preferences, medical, family, and social history, when determining their risk for cancer and the implications of MCED. As a field, we must stay abreast of emerging data to better understand how to advocate the fair and appropriate incorporation of MCED tests and their downstream implications into our practice.

## Author Contributions

**Elena Kennedy**, design, conduct, analysis, writing of the manuscript, final approval of the manuscript; **Greg Durm**, design, writing of the manuscript, final approval of the manuscript; **Janice L. Farlow**, design, conduct, analysis, writing of the manuscript, final approval of the manuscript.

## Disclosures

### Competing interests

GD: None related to MCED, however, Dr. Durm has grant and research support from Merck, BMS, and Astra Zeneca, and has honoraria from Dava Oncology, Curio Science, and Astra Zeneca.

### Funding source

None.

## Supporting information

Supporting Information.
